# Light and temperature records of the seawater associated with southern elephant seal dives during foraging trips in South Atlantic and Pacific Oceans

**DOI:** 10.3897/BDJ.11.e101284

**Published:** 2023-04-26

**Authors:** Elena B. Eder, Marcos Zárate, Mirtha N. Lewis

**Affiliations:** 1 Centre for the Study of Marine Systems, Centro Nacional Patagónico (CESIMAR-CENPAT-CONICET), Puerto Madryn, Argentina Centre for the Study of Marine Systems, Centro Nacional Patagónico (CESIMAR-CENPAT-CONICET) Puerto Madryn Argentina

**Keywords:** southern elephant seals, Southern Ocean, sea water light-temperature records

## Abstract

**Background:**

The dataset comprises geolocalised records of dive and surface interval durations, light level and temperature of the seawater during the post-resting and post-moulting tracks of 13 immature southern elephant seals, *Miroungaleonina*. It describes an unpublished open access version of the original data with records of light level and temperature of the water column using the Darwin Core standard (DwC) through ArOBIS, guaranteeing compliance with the FAIR principles, encompassing a wide time scale (2005, 2006 and 2007) and geographic range in the South Atlantic and Pacific Oceans (South West [-58.75, -81.29], North East [-37.60, -28.65]). Seals were simultaneously equipped with affordable light–temperature loggers (LTLs) and satellite tags. The LTLs recorded light level and temperature of the water column at 30-s intervals during dives and light–time records were applied to estimate dive parameters of diurnal records from 06:00 to 17:00 h, since movements up and down the water column are reflected by changes in light level. For that, the minimum light level reached at the surface of a dive was determined experimentally with diurnal dive simulations at sea using the LTLs devices before deployment. The dataset also includes variation of light and temperature of records between 17:00 to 06:00 h. Data can be used to identify temperature changes associated with seawater masses as drivers of the distribution of other taxa of interest and variation of light level in the seawater (light attenuation) could be linked to concentrations of phytoplankton assemblages as an index of primary productivity.

**New information:**

This dataset provides unpublished data of the duration of dives and surface intervals and associated records of light level and temperature variations along the movements throughout the seawater of 13 immature southern elephant seals in the Southern Hemisphere. The location data were generated by satellite tags and the light and temperature data were recorded with light-temperature loggers (LTLs), both devices deployed on individuals simultaneously and uploaded following the Darwin Core standard and compliance with the FAIR principles.

## Introduction

Elephant seals belong to the clade of Pinnipeds, a taxonomic group of mammals adapted to marine life that, however, conserve a dependence on the terrestrial environment, amongst other characteristics. During their annual cycle, seals predictably and synchronously alternate brief ashore periods dedicated to breeding, moulting or resting at very high-fidelity sites, with longer periods of exclusively marine feeding (post-breeding, post-moulting or post-resting trips) occupying more than 90% of the annual cycle time (Lewis and Eder 2021). Amongst the characteristics of the southern species (*Miroungaleonina)* when foraging at sea, is that they continuously dive to depths between 200 and 700 m (up to 2000 m+), for periods of 20 to 30 min (up to 120 min), making regularly 50 to 80 dives per day during this time (Lewis and Eder 2021), while surfacing briefly to replenish their oxygen stores between consecutive long dives (Hindell et al. 2016). Southern elephant seals have a circumpolar distribution in the Southern Hemisphere and they are wide-ranging foragers that undertake long migrations of thousands of kilometres at sea over broad geographic and oceanographic regions, spending significant time in highly productive water masses (fronts, currents, marginal pack ice zones etc) (Campagna et al. 2006, Campagna et al. 2007, Bailleul et al. 2007, Biuw et al. 2010, Mcintyre et al. 2012, McIntyre et al. 2011, Tosh et al. 2015, Páez-Rosas et al. 2018). Due to these characteristics, these marine mammals can be effective bio-platforms to collect data on marine environmental variables when they are equipped with miniaturised biologging devices during their foraging trips at sea (Hindell et al. 2016). In this sense, they can provide valuable data of the water column of broad geographic regions, from continental margins to deep basins, reaching areas where oceanographic campaigns are not often able to attain.

The dataset presented in this paper covers locations of 13 immature southern elephant seals during their post-resting and post-moulting feeding trips at sea and provides unpublished data of the duration of dives and surface intervals and associated light level and temperature variations along the movements throughout the seawater recorded by LTLs deployed on the individuals, following the DwC standard and compliance with the FAIR principles (Wilkinson et al. 2016). The study that originated the dataset was aimed to compare the duration diving pattern of juvenile individuals instrumented with LTLs, to assess if diving effort, determined by extended dives and long surface intervals, differed between contrasting foraging locations in terms of associated bathymetry and to hypothesise how these conditions may impact on juvenile foraging success and survival (Eder et al. 2011). Given that the dataset reported environmental data from the seawater covering a wide temporal (2005-2007) and geographic (South Atlantic and Pacific Oceans) scale, the information provided could be valuable for associations with other taxa and concentrations of phytoplankton assemblages as an index of primary productivity (Behrenfeld and Boss 2003), amongst other purposes.

## Project description

### Title

Light and temperature records of the seawater associated with southern elephant seal dives during foraging trips in South Atlantic and Pacific Oceans

### Personnel

Elena Eder, Marcos Zarate and Mirtha Lewis

### Funding

Agencia Nacional de Promoción Científica y Tecnológica PICT 01- 11749 and Consejo Nacional de Investigaciones Científicas y Técnicas (CONICET) PIP 02462 Resolución 1123/03, Australian Antarctic Division and National Research Council of Argentina Ph.D. programme (CONICET). The publication of this data paper was supported by the Belgian Science Policy Office (BELSPO, contract n°FR/36/AN1/AntaBIS) in the Framework of EU-Lifewatch as a contribution the SCAR Antarctic biodiveristy portal (biodiversity.aq).

## Sampling methods

### Study extent

**Figure 1.**
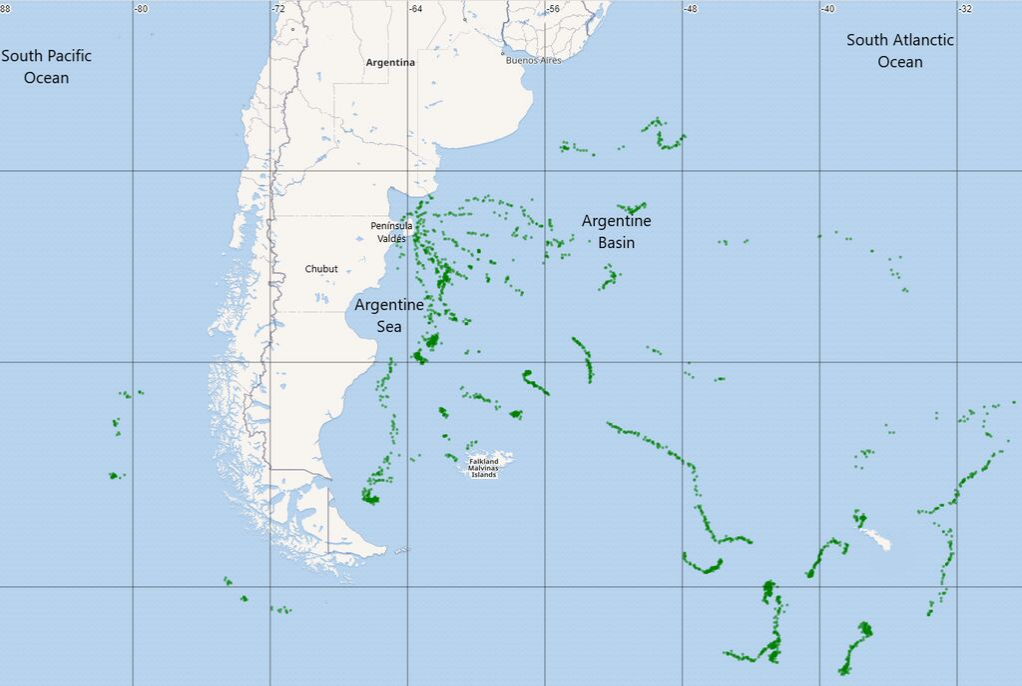
Study area with the locations of the associated data from the seawater during the feeding trips of the southern elephant seals. Green points represent locations where seawater light level and temperature data were collected.

The locations and associated data from the seawater (light level and temperature) during the feeding trips of the southern elephant seals encompassed an area between the South Atlantic and Pacific Oceans (South West [-58.75, -81.29], North East [-37.60, -26.54]). Fig. 1

### Sampling description

LTL devices (Platypus Engineering, Sydney, Australia) were deployed simultaneously with satellite tags (SPOT4/SPOT5; Wildlife Computers, Redmond, Washington) on 13 immature southern elephant seals from the Península Valdés colony, Patagonia, Argentina (42°45’S, 63°38’W). The setting and deployment protocols of instruments and their recovery have been described previously in Campagna et al. (2006), Campagna et al. (2007) and Eder et al. (2011). Data recorded encompass the post-resting and post-moulting feeding trips of individuals during 2005 (n = 4), 2005-2006 (n = 7) and 2007 (n = 2) seasons. Additionally, morphometric data of individuals taken during deployment and retrieval of the instruments and body mass estimates before and after feeding trips, are also provided. Standard length (SL, snout-tail length) and maximum girth (MG) were taken with a measuring tape to the nearest 0.5 cm, in ventral recumbency and body mass (EBM) was estimated using the equation in Bell et al. (1997) (EBM= 53.896 SL^1.063^ * MG^1.697^).

Estimation of dive durations and surface intervals

The variation of light registered by the LTLs (records were sampled at an interval of 30-s, expressed as hh/mm/ss), were used to estimate the dive durations and the surface intervals of dives, as vertical movements through the water column are reflected by changes in the light level (from maximum value at the surface = 250 arbitrary units, to total darkness in deep water = 2 arbitrary units). Simulations of dives at sea with the instruments under different conditions (different seasons, total, partial or no cloud cover and different orientations of the light sensor), determined a conservative minimum value of saturated light level of 190 units at the sea surface. The details of these simulations to estimate dive duration are described in Eder et al. (2011). According to this, dive durations and surface intervals of individuals were estimated, based on diurnal records between 06:00 and 17:00 h of the LTLs (local time; Eder et al. (2011)). Records from night or long dives during sunrise and sunset (hours of attenuated daylight) from the winter months were excluded for this analysis (Eder et al. 2011), but they were included in the dataset in order to provide the temperature variation.

Validation and correction of the temperature data

**Figure 2.**
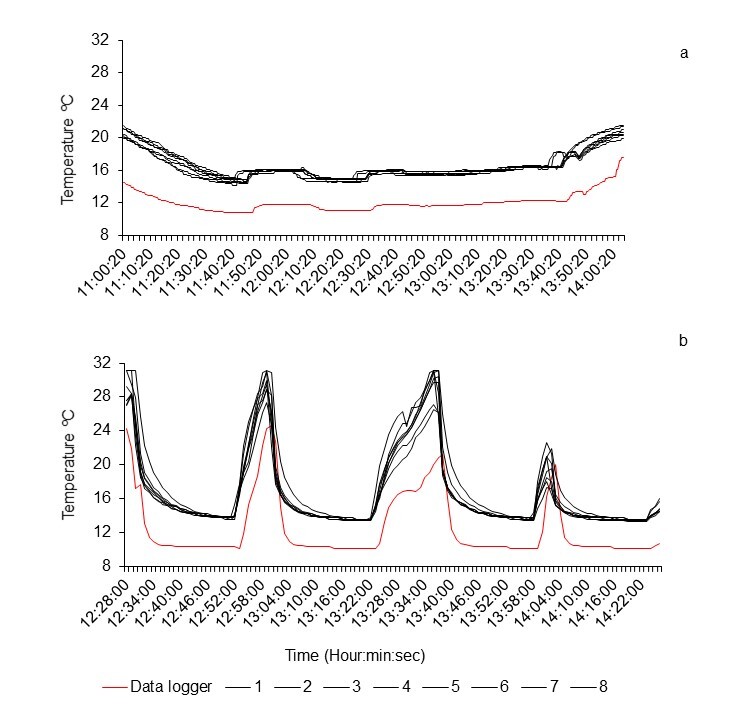
The LTLs (1-8) and the autonomous thermometer (data logger) temperature profiles during diving simulations at sea. a) From a boat (two dives), during a cloudy day, at a maximum depth of 35 m. b) From a local wharf (four dives with the devices orientated in the positions described in Eder et al. (2011)), during a clear day, at a maximum depth of 18.5 m.

**Figure 3.**
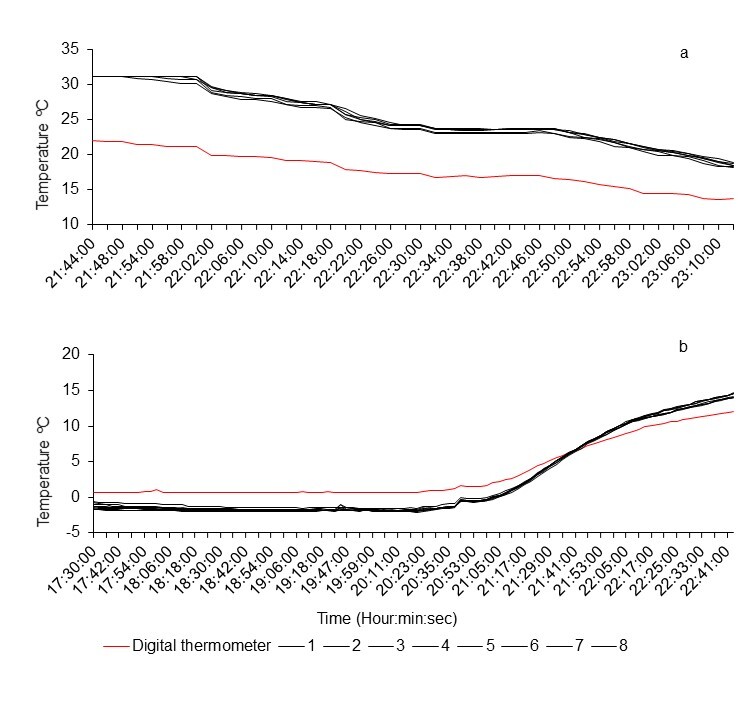
LTLs (1-8) temperature profiles during the gradual rise (a) and decrease (b) in the bath temperature at lab.

**Figure 4.**
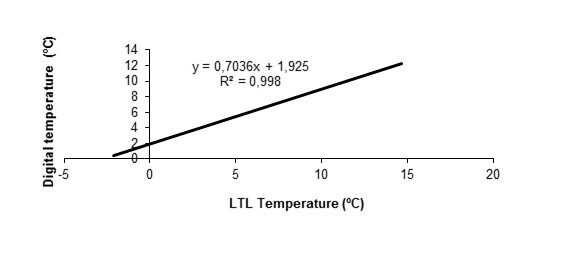
Adjusted function to correct the temperatures recorded by the LTL.

**Figure 5.**
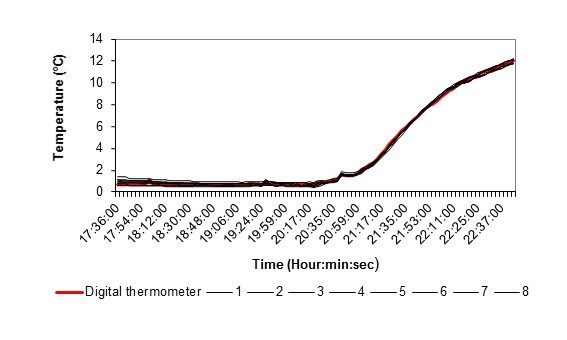
Overlapped temperature profiles of the digital thermometer and the LTLs (1-8) after correction.

The LTLs measure seawater temperature with a resolution of approximately 0.2ºC, over a range of -12 to 31ºC. However, some devices showed temperature records from -1.1 to 31.1ºC, which may not be appropriate values for the Argentine Sea and the adjacent Oceanic Basin. For this reason, the values of the devices were validated against an autonomous thermometer (Optic StowAway Temp) activated during the diving simulations and a digital thermometer during laboratory tests, to correct the temperature values of the LTLs. Fig. 2 shows the temperature profile recorded by the devices and the autonomous thermometer during the diving simulations detailed in Eder et al. (2011). During these simulations, the recorded temperatures never fell below 10ºC and the differences between the records of the LTL and the autonomous thermometer, under different conditions, was 4.4 ± 0.8ºC and 4.4 ± 2.3ºC. Fig. 3 shows the profile of the LTL and the digital thermometer records during exposure to low and high temperatures in the laboratory. In these experiences, the temperature difference was 2.1 ± 0.6ºC when the exposure was from 0.7 to 12ºC, 7.4 ± 1.4 when the temperature was 22 to 13.7ºC and 6.3 ± 1.9 when exposed to 31.06 and 22ºC. As can be seen in Figs 2, 3, the LTL temperature records behave differently at low and relatively high values. At temperatures below 6ºC, the records tended to underestimate the temperature values, although the difference is somewhat less than at temperatures greater than 6ºC, when the records tended to overestimate the values until reaching the upper limit of the range that the device can measure. Given this irregular behaviour of the temperature records of the LTL, the temperature data obtained in the laboratory were used to obtain a general adjustment function to correct the entire range of temperatures registered by the devices (Fig. 4). Fig. 5 shows the temperatures recorded with the digital thermometer and the temperatures of the equipment once the correction was applied.

### Quality control

All records were validated. The coordinates were validated using the **check_onland()** function of the obistools package to verify if there are points on land. Although the dataset has only one taxon, match_taxa() was used to determine if the taxonomic name is valid. All scientific names were checked for typos and matched to the species information backbone of Worlds Register of Marine Species (http:// marinespecies.org/) and LSID were assigned to taxon as scientificNameID. The original date data columns were converted with the OpenRefine tool to ISO 8601 format, which was assigned to the eventDate field of the Dwc standard. To check the consistency of the eventID and parentEventID fields, the check_eventids() function was used.

## Geographic coverage

### Description

The locations during the feeding trips of the southern elephant seals encompassed an area between the South Atlantic and Pacific Oceans (South West [-58.75, -81.29], North East [-37.60, -28.65]). Most of the records are located within the well-known foraging areas of southern elephant seals from the Península Valdés colony. These include the continental shelf, an area of less than 200 m in depth that extends 300-400 km east from the coast and is characterised by mixed coastal and stratified waters (Campagna et al. 2006), the shelf break, where the Malvinas Current carry cold sub-Antarctic waters north from the Antarctic Circumpolar Current and the encounter with low-salinity shelf water originates a shelf-break front associated with temperature and salinity gradients and increased primary productivity (McGovern et al. 2022), and the deep Argentine Basin (6000 m), where the warm-salty subtropical waters, carried southwards by the Brazil Current, meet the cold-fresh subpolar waters carried northwards by the Malvinas Current, producing the Brazil-Malvinas Confluence, characterised by increased primary productivity, large temperature gradients and intense mesoscale eddy activity (Campagna et al. 2006, McGovern et al. 2022).

## Taxonomic coverage

### Taxa included

**Table:** 

Rank	Scientific Name	
class	Mammalia	
order	Carnivora	
family	Phocidae	
genus	*Mirounga*	
kingdom	*Miroungaleonina* (Linnaeus, 1758)	

## Temporal coverage

**Single date:** ; .

### Notes

Data recorded encompass the post-resting feeding trips of four individuals from the 2005 season, the post-moulting feeding trips of seven individuals from the 2005-2006 season and the post-moulting feeding trips of two individuals from the 2007 season (Zarate and Eder 2022). Date ranges (and tracking period in days) are detailed below:

MAR3: 2005-07-29/2005-11-14 (108)

BK6: 2005-08-02/2005-11-02 (92)

SI4: 2005-08-08/2005-10-22 (75)

SyS2: 2005-08-15/2005-11-11 (88)

RON6: 2005-12-07/2006-02-17 (72)

LIN3: 2005-12-11/2006-07-11 (216)

SUR1: 2005-12-13/2006-02-07 (56)

RUS2: 2005-12-13/2006-02-23 (72)

BUC10: 2005-12-16/2006-05-06 (141)

FAR11: 2005-12-17/2006-04-22 (126)

PT7: 2005-12-19/2006-04-23 (125)

2LID: 2007-01-04/2007-06-27 (174)

1LID: 2007-01-06/2007-07-29 (204)

## Usage licence

### Usage licence

Creative Commons Public Domain Waiver (CC-Zero)

## Data resources

### Data package title

Light and temperature records of the seawater associated with southern elephant seal dives during foraging trips in South Atlantic and Pacific Oceans.

### Resource link

https://www.gbif.org/dataset/fa11b646-142c-49ec-915c-8b94b9d4bba3

### Alternative identifiers

https://www.movebank.org/cms/webapp?gwt_fragment=page=studies,path=study2706892261

### Number of data sets

2

### Data set 1.

#### Data set name

Light and temperature records of the seawater associated with southern elephant seal dives during foraging trips in South Atlantic and Pacific Oceans.

#### Data format

Darwin core

#### Character set

UTF-8

#### Download URL

https://arobis.cenpat-conicet.gob.ar:8081/resource?r=ses-light-temperature

#### Description

This dataset describes an unpublished open access version of the original data with records of light level and temperature of the water column using the Darwin Core standard (DwC) through ArOBIS, guaranteeing compliance with the FAIR principles. The LTLs recorded light level and temperature of the water column at 30-s intervals during dives and light–time records were applied to estimate dive parameters of diurnal records from 06:00 to 17:00 h, since movements up and down the water column are reflected by changes in light level. For that, the minimum light level reached at the surface of a dive was determined experimentally with diurnal dive simulations at sea using the LTLs devices before deployment. As variation of temperature in the water column can be associated with the local distribution of other taxa of interest and variation of light level in the water column (light attenuation) could be linked to concentrations of phytoplankton assemblages as an index of primary productivity, the dataset can be of useful interest. This dataset also includes variation of light and temperature of records between 17:00 to 06:00 h. The dataset encompasses a wide time scale (2005, 2006 and 2007) and covers a wide geographic range in the South Atlantic and Pacific Oceans (South West [-58.75, -81.29], North East [-37.60, -28.65]).

If you have any questions regarding this dataset, please do not hesitate to contact us via the contact information provided in the metadata or via eder@cenpat-conicet.gob.ar.

**Data set 1.** 

Column label	Column description
eventID	an identifier for the set of information associated with an Event (something that occurs at a place and time). This may be a global unique identifier or an identifier specific to the dataset.
parentEventID	An identifier for the broader Event that groups this and potentially other Events.
type	the event type information is provided in this column.
eventDate	the date or interval during which an Event occurred.
eventTime	the time or interval during which an Event occurred.
decimalLatitude	the geographic latitude (in decimal degrees, using the spatial reference system given in geodeticDatum) of the geographic centre of a Location. Positive values are north of the Equator, negative values are south of it. Legal values lie between -90 and 90, inclusive.
decimalLongitude	the geographic longitude (in decimal degrees, using the spatial reference system given in geodeticDatum) of the geographic centre of a Location. Positive values are east of the Greenwich Meridian, negative values are west of it. Legal values lie between -180 and 180, inclusive.
minimumDepthInMetres	minimum depth during event in metres.
maximumDepthInMetres	maximum depth during event in metres.
institutionCode	institution code.
datasetName	dataset name.
occurrenceID	an identifier for the Occurrence/specimen.
basisOfRecord	the specific nature of the data record.
occurrenceStatus	a statement about the presence or absence of a Taxon at a Location.
scientificName	scientific name.
scientificNameID	marinespecies.org taxon number.
scientificNameAuthorship	the authorship information for the scientificName formatted according to the conventions of the applicable nomenclaturalCode.
sex	sex.
kingdom	the full scientific name of the kingdom in which the taxon is classified.
phylum	the full scientific name of the phylum in whch the taxon is classified.
class	the full scientific name of the class in which the taxon is classified.
order	the full scientific name of the order in which the taxon is classified.
family	the full scientific name of the family in which the taxon is classified.
genus	the full scientific name of the genus in which the taxon is classified.
taxonRank	the taxonomic rank of the most specific name in the scientificName.
measurementType	the nature of the measurement, fact, characteristic or assertion.
measurementTypeID	a machine-readable URI or DOI reference describing the (version of the) classification system itself.
measurementValue	the value of the measurement, fact, characteristic or assertion.
measurementValueID	if available, a machine-readable URI describing the habitat class in “measurementValue”.
measurementUnit	the units associated with the measurementValue.
measurementUnitID	if available, a machine-readable URI describing the measurement in “measurementUnit”.

### Data set 2.

#### Data set name

Light and temperature records of the seawater associated with southern elephant seal dives during foraging trips in South Atlantic and Pacific Oceans.

#### Data format

tsv

#### Character set

UTF-8

#### Download URL

https://www.movebank.org/cms/webapp?gwt_fragment=page=studies,path=study2706892261

#### Description

The dataset contains records of locations of 13 immature southern elephant seals, Miroungaleonina, during their post-resting and post-moulting feeding trips at sea. The dataset encompasses a wide time scale (2005, 2006 and 2007) and covers a wide geographic range in the South Atlantic and Pacific Oceans (South West [-58.75, -81.29], North East [-37.60, -28.65]).

**Data set 2.** 

Column label	Column description
animalID	A unique identifier for the deployment of a tag on animal.
Timestamp	The timestamp when the tag deployment started. Format: yyyy-MM-dd'T'HH:mm:ss'Z'
Location lat	The geographic latitude of the location where the animal was. Units: decimal degrees, WGS84 reference system.
Location long	The geographic longitude of the location where the animal was. Units: decimal degrees, WGS84 reference system.
Comments	Additional information about the tag deployment that is not described by other reference data terms.
